# Smartphone-Based Indoor Localization within a 13th Century Historic Building

**DOI:** 10.3390/s18124095

**Published:** 2018-11-22

**Authors:** Toni Fetzer, Frank Ebner, Markus Bullmann, Frank Deinzer, Marcin Grzegorzek

**Affiliations:** 1 Faculty of Computer Science and Business Information Systems, University of Applied Sciences Würzburg-Schweinfurt, 97074 Würzburg, Germany; frank.ebner@fhws.de (F.E.); markus.bullmann@fhws.de (M.B.); frank.deinzer@fhws.de (F.D.); 2 Institute of Medical Informatics, University of Lübeck, 23538 Lübeck, Germany; grzegorzek@imi.uni-luebeck.de

**Keywords:** indoor localization, Wi-Fi, PDR, sensor fusion, smartphone, particle filter, sample impoverishment, estimation, historic buildings, navigation mesh

## Abstract

Within this work we present an updated version of our indoor localization system for smartphones. The pedestrian’s position is given by means of recursive state estimation using a particle filter to incorporate different probabilistic sensor models. Our recently presented approximation scheme of the kernel density estimation allows to find an exact estimation of the current position, compared to classical methods like weighted-average. Absolute positioning information is given by a comparison between recent Wi-Fi measurements of nearby access points and signal strength predictions. Instead of using time-consuming approaches like classic fingerprinting or measuring the exact positions of access points, we use an optimization scheme based on a set of reference measurements to estimate a corresponding Wi-Fi model. This work provides three major contributions to the system. The most essential contribution is the novel state transition based on continuous walks along a navigation mesh, modeling only the building’s walkable areas. The localization system is further updated by incorporating a threshold-based activity recognition using barometer and accelerometer readings, allowing for continuous and smooth floor changes. Within the scope of this work, we tackle problems like multimodal densities and sample impoverishment (system gets stuck) by introducing different countermeasures. For the latter, a simplification of our previous solution is presented for the first time, which does not involve any major changes to the particle filter. The goal of this work is to propose a fast to deploy localization solution, that provides reasonable results in a high variety of situations. To stress our system, we have chosen a very challenging test scenario. All experiments were conducted within a 13th century historic building, formerly a convent and today a museum. The system is evaluated using 28 distinct measurement series on four different test walks, up to 310 m length and 10 min duration. It can be shown, that the here presented localization solution is able to provide a small positioning error, even under difficult conditions and faulty measurements. The introduced filtering methods allow for a real fail-safe system, while the optimization scheme enables an on-site setup-time of less then 120 min for the building’s 2500 m^2^ walkable area.

## 1. Introduction

Setting up a reliable localization solution for a building is a challenging and time-consuming task, especially in environments that are not built with localization in mind or do not provide any wireless infrastructure or even both. Such scenarios are of special interest when old or historical buildings serve a new purpose such as museums, shopping malls or retirement homes. In terms of European architecture, the problems emanating from these buildings worsen over time.

In the scope of this work, we deployed an indoor localization system to a 13th century building. The first 300 years, the building was initially used as a convent, and, after that, had different functions ranging from a granary to an office for Bavarian officials. Over time, the building underwent major construction measures and was extended several times. Since 1936, the 2500 m^2^ building acts as a museum of the medieval town Rothenburg ob der Tauber [1], Germany.

Such buildings are often full of nooks and crannies, what makes it hard for dynamical models using any kind of pedestrian dead reckoning (PDR). Here, the error accumulates not only over time, but also with the number of turns and steps made [2]. There is also a higher probability of detecting a wrong turn, what can cause the position estimation to lose track or getting stuck within a demarcated area. Thus, this paper presents a continuous movement model using a three-dimensional navigation mesh based on triangles. In addition, a threshold-based activity-recognition is used to allow for smooth floor changes.

In localization systems using a sample based density representation, like particle filters, aforementioned problems can further lead to more advanced problems like sample impoverishment [3] or multimodalities [4]. Sample impoverishment refers to a situation, in which the filter is unable to sample enough particles into proper regions of the building, caused by a high concentration of misplaced particles. Within this work we present a simple yet efficient method that enables a particle filter to fully recover from sample impoverishment. We also use an approach for finding an exact estimation of the pedestrian’s current position by using an approximation scheme of the kernel density estimation (KDE) [5].

Many historical buildings, especially bigger ones like castles, monasteries or churches, are built of massive stone walls and have annexes from different historical periods out of different construction materials. This makes it more challenging to ensure good radio coverage of the entire building, especially for technologies using received signal strengths indications (RSSI) from Wi-Fi or Bluetooth. For methods requiring environmental knowledge, like signal strength prediction models, the high signal attenuation between different rooms causes further problems. Many unknown quantities, like the walls definitive material or thickness, make it expensive to determine important parameters, e.g., the signal’s depletion over distance. Additionally, many of these approaches are based on a line-of-sight assumption. Thus, the performance will be even more limited due to the irregularly shaped spatial structure of such buildings. Our approach tries to avoid those problems using an optimization scheme based on a set of Wi-Fi reference measurements. We distribute a set of small (2.8 cm × 3.5 cm) and cheap (≈$10) Wi-Fi beacons over the whole building to ensure a reasonable coverage and instead of measuring their position and necessary parameters, we use our optimization scheme, initially presented in [6].

This optimization scheme is able to compensate for wrongly measured access point positions, inaccurate building plans or other knowledge necessary for the Wi-Fi component. Of course, this could be solved by re-measuring the building, however this is a very time-consuming process requiring specialized hardware and surveying engineer. Depending on the size of the building, such a complex and time-consuming process is contrary to most costumers expectations of a fast to deploy solution. In addition, this is not just a question of initial effort, but it is also problematic for buildings under monumental protection, not allowing larger construction measures. That is why the compact Wi-Fi beacons are a reasonable alternative to conventional access points for localization. The access points of a regular Wi-Fi network infrastructure are mostly mounted to the ceilings of the building to presume a cost efficient setting receiving the highest possible coverage. However, this usually requires new cabling, e.g., an extra power over Ethernet connection. In contrast, the beacons can simply be plugged into already existing power outlets and due to their low price they can be distributed in large quantities, if necessary. In the here presented scenario, the beacons do not establish a wireless network and thus serve only to provide signal strengths.

To sum up, this work presents an updated version of the winning localization system of the smartphone-based competition at IPIN 2016 [4,7], including the improvements and newly developed methods that have been made since then [3,5,6,8]. This is the first time that all these previously acquired findings have been fully combined and applied simultaneously. During the here presented update, the following contributions will be presented and added to the system:

- The pedestrian’s movement is modelled in a more realistic way using a navigation mesh, generated from the building’s floor plan. This only allows movements that are actually feasible, e.g., no walking through walls. Compared to the gridded graph structure we used before [8], the mesh allows continuous transitions and reduces the required storage space drastically.
- To enabled more smooth floor changes, a threshold-based activity recognition using barometer and accelerometer readings is added to the state evaluation process of the particle filter. The method is able to distinguish between standing, walking, walking up and walking down.
- To address the problem of sample impoverishment in a wider scope, we present a simplification of our previous method [3]. This reduces the overhead of adapting an existing system to the proposed method and allows to incorporate it directly to the state transition of any approach, using a general particle filter methodology.

The goal of this work is to propose a fast to deploy localization solution, that provides reasonable results in a high variety of situations. However, many state-of-the-art solutions tend to be evaluated within office or faculty buildings, offering a modern environment and well described infrastructure. Consequently, we believe that by utilizing our localization approach to such a challenging scenario, it is possible to prove those characteristics. To initially set up the system we only require a blueprint to create the floor plan, some Wi-Fi infrastructure, without any further information about access point positions or parameters, and a smartphone carried by the pedestrian to be localized. The existing Wi-Fi infrastructure can consist of the aforementioned Wi-Fi beacons and/or already existing access points. The combination of both technologies is feasible. Nevertheless, the museum considered in this work has no Wi-Fi infrastructure at all, not even a single access point. Thus, we distributed a set of 42 beacons throughout the complete building by simply plugging them into available power outlets. In addition to evaluating the contributions and the overall performance of the system, we have carried out further experiments to determine the performance of our Wi-Fi optimization in such a complex scenario as well as a detailed comparison between KDE-based and weighted-average position estimation.

## 2. Related Work

We consider indoor localization to be a time-sequential, non-linear and non-Gaussian state estimation problem. Such problems are often solved using Bayesian filters, which update a state estimation recursively with every new incoming measurement. A powerful group of methods to obtain numerical results for this approach are particle filter.

In context of indoor localization, particle filter approximate a probability distribution describing the pedestrian’s possible whereabouts by using a set of weighted random samples (particles). Here, new particles are drawn according to some importance distribution, often represented by the state transition, which models the dynamics of the system. Those particles are then weighted by the state evaluation given different sensor measurements. A resampling step is deployed to prevent that only a small number of particles have a significant weight [9]. Most localization approaches differ mainly in how the transition and evaluation steps are implemented and the sensors are incorporated [10,11,12,13].

The system’s dynamics describe a pedestrian’s potential movement within the building. This can be formulated as the question *“Given the pedestrian’s current position and heading are known, where could he be after a certain amount of time?”*. Obviously, the answer to this question depends on the pedestrian’s walking behavior, any nearby architecture and thus the building’s floor plan. Assuming the pedestrian to walk almost straight towards his current heading with a known, constant walking speed, the most basic form of state transition simply rejects all movements, where the line-of-sight between current position and potential destination is blocked by an obstacle [14,15]. Despite its simplicity, this approach suffers from several drawbacks. The intersection-test can be costly, depending on the number of used particles and the complexity of the building. Furthermore, it is limited mainly to 2D transitions within the plane. Smooth 3D transitions, like walking stairs, would require much more complex intersection tests [16].

To overcome both limitations, the building’s floor plan can be used to derive a graph-based structure, like voronoi diagrams or fixed-distance grids, moving all costly intersection tests into a one-time offline phase [8,13]. Hereafter, graph-based random walks along the created data-structure can be used as a fast transition approximation. Smooth transitions in 3D space can be achieved by generating nodes and edges along stairs and elevators. Furthermore, the nodes can be used to store additional information, like their distance towards a pedestrian’s desired destination. Such information can be included during the transitions step, e.g., increasing the likelihood of all potential movements that approach this destination [8].

However, the graph-based approach also imposes some potential issues. When using a gridded graph, the spacing between adjacent nodes directly represents the transition’s accuracy. Likewise, the amount of required memory to represent the floor plan scales about quadratically with this spacing. Even though nodes/edges are only created for actually walkable areas (like a sparse cube), large buildings require millions of nodes and might not fit into memory at once. Furthermore, (large) outdoor regions between adjacent buildings require unnecessarily large amounts of memory to be modeled [16]. While voronoi diagrams have the ability to mitigate this issue to some degree, they usually suffer from reduced accuracy for large open spaces, as many implementations only use the edges to estimate potential movements [13].

We therefore present a novel technique based on continuous walks along a navigation mesh. Like the graph, the mesh, consisting of triangles sharing adjacent edges, is created once during an offline phase, based on the building’s 3D floor plan. Using large triangles reduces the memory footprint dramatically (a few megabytes for large buildings) while still increasing the quality (triangle-edges directly adhere to architectural-edges) and allows for truly continuous transitions along the surface spanned by all triangles.

To be able to go into the third dimension, cf. walking continuously along stairs, many localization system consider using a barometer [17,18,19]. As absolute pressure readings highly differ between season, time of day and sometimes even individual areas of the building, approaches considering the relative change of pressure are mostly preferred. For example, this can be done by initializing with a zero pressure at the beginning of every walk or using some sliding window [20,21]. Such approaches can then be easily integrated into the movement model, as they provide continuous updates with every incoming barometer reading. However, the accuracy then often depends on the quality of the sensor’s (raw) measurements. Using smoothing statistics like Kalman filtering or a moving average, might thus be valid options to further improve the results. Thanks to the underlying mesh another possibility to provide continues floor changes can be considered: the pedestrian’s current activity. This is done by assigning different labels to the triangles of the mesh, e.g., stair, floor or elevator. Depending on the recognized activity, the system is now able to allow or restrict the movement in certain areas of the building. A probabilistic model for incorporating this into the particle filter, will be presented within this work.

In recent years, many different activity recognition approaches could be presented for wearable sensors [22]. They occur in a wide variety of scenarios, such as in sports or in the health sector. As modern smartphones become more and more powerful, classical approaches of pattern recognition can now be adapted directly. Nevertheless, in context of detecting activities in indoor environments, such approaches might be to much of a good thing. For example, authors [23] provide very promising results, but their approach requires an extensive training-phase using a set of previously recorded training data. Acquiring such data can not only be time-consuming, but often opens the need for a high diversity, to model multiple different movement patterns and thus prevent an overadaption of the classification to a small set of testers. Because of this, we present a threshold-based activity recognition, that can cover a general setting without much prior knowledge. This is a very straightforward approach, what can be found especially in early literature on activity detection using wearable sensors [24,25,26]. In contrast to recent state-of-the-art methods, which try to incorporate many different activities, we are only interested in finding four activities, namely standing, walking, walking up and down. This limitation allows to consider the present scenario in the best possible way. It should be noted, that our approach cannot necessarily keep up with the more advanced methods mentioned above, but it shows suitable results in the context of indoor localization.

Most smartphone-based systems for indoor localization are using received signal strength indications (RSSI) given by Wi-Fi or Bluetooth as a source for absolute positioning information. At this, one can mainly distinguish between fingerprinting and signal strength prediction model based solutions [6]. Indoor localization using Wi-Fi fingerprints was first addressed by [27]. During a one-time offline-phase, a multitude of reference measurements are conducted. During the online-phase the pedestrian’s location is then inferred by comparing those prior measurements against live readings. Based on this pioneering work, many further improvements where made within this field of research [28,29,30]. However, despite a very high accuracy up to 1 m, classic fingerprinting approaches suffer from tremendous setup- and maintenance times. For this reason, some alternative approaches were presented to speed up the offline-phase. In [31] the positions of recorded references are interpolated between the start and end of some reference path, based on the pedestrians gait cycle. Unrecorded positions are then obtained using the flood fill algorithm. However, for old buildings with many nooks and crannies this might cause problems as the RSSI can differ highly within a few meter, especially in the entrance area of thick-walled rooms. This could open the need for more advanced map interpolation methods or a higher number and density of reference paths to walk. Another often considered alternative is using robots instead of human workforce [32,33], still this seems not to be a valid option for old buildings with limited accessibility for robots due to uneven grounds and small stairs.

Signal strength prediction models are a well-established field of research to determine signal strengths for arbitrary locations by using an estimation model instead of real measurements. While many of them are intended for outdoor and line-of-sight purposes [34,35], they are often applied to indoor use-cases as well [6,36]. Besides their solid performance in many different localization solutions, a complex scenario requires an equally complex signal strength prediction model. As described in Section 1, historical buildings represent such a scenario and thus the model has to take many different constraints into account. An example is the wall-attenuation-factor model [37]. It introduces an additional parameter to the well-known log-distance model [38], which considers obstacles between (line-of-sight) the access point (AP) and the location in question by attenuating the signal with a constant value. Depending on the use-case, this value describes the number and type of walls, ceilings, floors etc. between both positions. For obstacles, this requires an intersection-test of each obstacle with the line-of-sight, which is costly for larger buildings. Thus [6] suggests to only consider floors/ceilings, which can be calculated without intersection checks and allows for real-time use-cases running on smartphones.

To further reduce the setup-time, authors [39] introduces an approach that works without any prior knowledge. They use a genetic optimization algorithm to estimate the parameters for a signal strength prediction, including access point positions, and the pedestrian’s locations during the walk. The estimated parameters can be refined using additional walks. Within this work we present a similar optimization approach for estimating the AP’s location in 3D. However, instead of taking multiple measuring walks, the locations are optimized based only on some reference measurements, further decreasing the setup-time. Additionally, we will show that such an optimization scheme can partly compensate for the above abolished intersection-tests.

Besides well chosen probabilistic models, the system’s performance is also highly affected by handling problems which are based on the nature of a particle filter. They are often caused by restrictive assumptions about the dynamic system, like seen from the aforementioned problem of sample impoverishment. The authors of [40] handled the problem by using an adaptive number of particles instead of a fixed one. The key idea is to choose a small number of samples if the distribution is focused on a small part of the state space and a large number of particles if the distribution is much more spread out and requires a higher diversity of samples. The problem of sample impoverishment is then addressed by adapting the number of particles dependent upon the system’s current uncertainty.

In practice, sample impoverishment is often a problem of environmental restrictions and system dynamics. Therefore, the method above fails, since it is not able to propagate new particles into the state space due to environmental restrictions e.g., walls or ceilings. In [3] we deployed an interacting multiple model particle filter (IMMPF) to solve sample impoverishment in such restrictive scenarios. We combine two particle filter using a non-trivial Markov switching process, depending upon the Kullback-Leibler divergence between both. However, deploying an IMMPF is in many cases not necessary and produces additional processing overhead. Thus, a much simpler, but heuristic method is presented within this paper.

Finally, as the name recursive state estimation says, it requires to find the most probable state within the state space, to provide the “best estimate” of the underlying problem. In the discrete manner of a particle representation this is often done by providing a single value, also known as sample statistic, to serve as a best guess [41]. Examples are the weighted-average over all particles or the particle with the highest weight [42]. However, in complex scenarios like a multimodal representation of the posterior, such methods fail to provide an accurate statement about the most probable state. Thus, in [5] we present a approximation scheme of kernel density estimates (KDE). Recovering the probability density function using an efficient KDE algorithm yields a promising approach to solve the state estimation problem in a more profound way.

## 3. Recursive State Estimation

We consider indoor localization to be a time-sequential, non-linear and non-Guassian state estimation problem. The filtering equation to calculate the posterior is given by the recursion

(1) $$\begin{array}{c} p(q_{t}|o_{1:t})\propto \\ \underset{\mathrm{evaluation}}{\underset{︸}{p(o_{t}|q_{t})}}\int \underset{\mathrm{transition}}{\underset{︸}{p(q_{t}|q_{t-1},o_{t-1})}}\underset{\mathrm{recursion}}{\underset{︸}{p\left(q_{t-1}|o_{1:t-1}\right)dq_{t-1}}} \end{array},$$

where $q_{t}$ is the hidden state and $o_{t}$ provides the corresponding observation vector at time *t*. As realization of (1) we use the well-known CONDENSATION particle filter [43]. Here, the transition is used as proposal distribution and a resampling step is utilized to handle the phenomenon of weight degeneracy.

The state q is given by

(2) q=(x,y,z,θ),x,y,z,θ∈R,

where x,y,z represent the position in 3D space and θ is the user’s current (absolute) heading. In context of particle filtering, a particle is thus a weighted representation of one possible state q. The observation vector is defined as

(3) $$o=(s_{\mathrm{wifi}},\Delta \theta ,n_{\mathrm{steps}},\Omega ).$$

Here, $s_{\mathrm{wifi}}$ contains the signal strength measurements of all access points currently visible to the phone. Δθ provides the relative angular change and $n_{\mathrm{steps}}$ the number of steps since the last filter-step. The result of a simple activity recognition using the phone’s barometer and acceleromter is given by Ω, which is one of: “standing”, “walking”, “walking up” or “walking down”.

## 4. Transition

**Figure 1:**
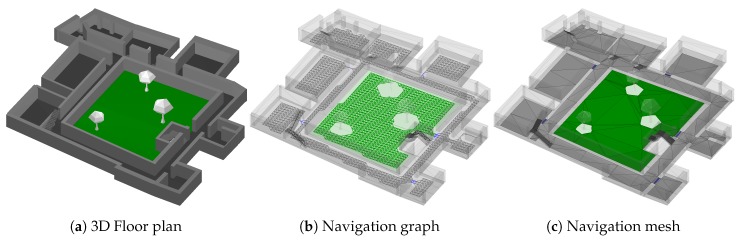
Floor plan (**a**) and automatically generated transition data structures (**b**,**c**) for the ground floor of the historic building ( 71 m × 53 m). To reach every nook and cranny, the generated graph (**b**) requires many nodes and edges. The depicted version uses a coarse node-spacing of 90 cm (1700 nodes) but barely reaches all doors and stairs. A navigation mesh generated for the same building required only 320 triangles (**c**) and reaches every corner within the building.

Within previous works, we used a graph of equidistant nodes (see Figure 1b) to model the building’s floor plan, representing the basis for the transition step [2,8]. It is created automatically, based on the building’s floor plan, which, in turn, results from manually tracing available blueprint pictures within our editing software.

The resulting graph equals a grid, where each node constitutes the center of a grid-cell. Each cell uses an empiric choice of 30 × 30 cm in size. The algorithm thus places a new cell every 30 cm, but only in regions that are actually walkable, and where the cell’s outline does not intersect any wall or other obstacles. As cells are equidistant and axis aligned for performance reasons, the algorithm works reasonably well for rectangular buildings, matching the graph’s coordinate system. For skewed floor plans, however, many periphery cells will intersect with walls and are thus omitted, reducing the quality of the representation. While smaller cells allow for a more accurate representation of the building, more cells are needed in total, increasing memory requirements for the smartphone. After placement, each cell is connected with their, up to 8, potential neighbors in the plane (left, right, top, bottom and diagonals). Those connections are only added, if the neighbor is actually available, and the connection itself does not intersect any obstacles. Doing so creates a walkable graph consisting of nodes and edges for each floor. These graphs are hereafter connected via stairs or elevators, to form the final, walkable data structure for the whole building. This allows for (semi-)random walks along the graph, modeling potential pedestrian movements. By assigning probabilities to each edge, using prior knowledge e.g., provided by sensors, the random walk along those weighted edges denotes the transition probability $p(q_{t}|q_{t-1},o_{t-1})$ [8].

Due to the equidistant spacing every 30 cm, the resulting graph is rather rigid and only well-suited for rectangular buildings. For more contorted buildings, like many historic ones, the node-spacing needs to be small, to reliably reach every door, stair and corner of the building. For demonstration, we used a 90 cm spacing within Figure 1b. As can be seen, the automatically generated grid is barely able to reach all places within the lower floors of the building, and fails to connect the upper floors reliably. While using smaller spacings remedies the problem, it requires huge amounts of memory: Up to hundred megabytes and millions of nodes and edges are realistic for larger buildings.

Because of both, required memory amounts and inaccuracies of the graph-based model depending on the spacing, we developed a new basis for the transition step, that is still able to answer $p(q_{t}|q_{t-1},o_{t-1})$, but has a much smaller memory footprint while representing the real floor plan more accurately. The new foundation is provided by well-known navigation meshes [44], where the walkable area is spanned by convex polygons. As the polygons are neither axis-aligned nor fixed in shape and size, they can accurately represent skewed architectures, where the grid-based approach suffers from aforementioned flaws. Another main idea behind navigation meshes is presented by shared outline edges between adjacent polygons. It thus is always possible to walk from one polygon into another, if they are adjacent. Similar to the graph-based approach, adjacent polygons denote some sort of walkable surface. However, while a graph restricts the movement to edges and nodes, the mesh allows for a true continues movement. This is achieved by having the freedom to walk to any position, under the condition that it resides within a polygon that is actually walkable from the starting position. Just as before, the navigation mesh can be automatically generated from the building’s floor plan, based on various algorithms [45,46]. In contrast to the graph, the number of polygons depend on the size and shape of the building as well as the used algorithm. Increasing the density of polygons intentionally, does not improve the accuracy, as would be the case for grid cells of the graph, due to aforementioned continues movement characteristic. This also removes the need of defining some kind of initial density for the mesh, like the spacing of the grid cells, what makes it more flexible.

Using variably shaped/sized elements instead of rigid grid-cells provides both, higher accuracy for reaching every corner, and a reduced memory footprint as a single polygon is able to cover arbitrarily large regions. For a rectangular room, the number of elements needed for the graph-based approach depends on the room’s size and the chosen spacing. The navigation mesh, however, only needs a single, four-sided polygon, to fully cover the rectangular shape of the room, independent of its size.

However, polygons impose several drawbacks on common operations used within the transition step, like checking whether a point is contained within some region. This is much more costly for polygons compared to axis-aligned, rectangular grid-cells. Such issues can be mitigated by using triangles instead of polygons, depicted within Figure 1c. Doing so, each element within the mesh has exactly three edges and a maximum of three neighbors. This usually requires some additional memory, as more triangles are need compared to polygons. For the example of the rectangular room, two adjacent triangles are required to form a rectangular shape. However, using triangles, operations such as aforementioned contains-check, can now easily be performed, e.g., by using barycentric coordinates, yielding noticeable speedups compared to polygons. This approach has established itself especially in the field of computer game development for solving pathfinding problems. A popular open-source library for creating navigation meshes in C++ is Recast [47].

This data structure yields room for various strategies to be applied within the transition step. The most simple approach uses an average pedestrian step size together with the number of detected steps $n_{\mathrm{steps}}$ and change in heading Δθ gathered from sensor observations $o_{t-1}$. Combined with previously estimated position ${\left(x,y,z\right)}^{T}$ and heading θ from $q_{t-1}$, including uncertainties for step-size S and turn-angle T, this directly defines new potential whereabouts $p(q_{t}|q_{t-1},o_{t-1})$:

(4) xt=xt−1︷oldpos.+nsteps·S︷distance·cos(θt)︷direction,T∼N(Δθ,σturn2)yt=yt−1.+nsteps·S·sin(θt),S∼N(70 cm,σstep2)zt=interpolatedθt=θt−1+T

with

$$n_{\mathrm{steps}},\Delta \theta \in o_{t-1},\;x_{t-1},y_{t-1},z_{t-1},\theta_{t-1}\in q_{t-1}\;\mathrm{and}\;x_{t},y_{t},z_{t},\theta_{t}\in q_{t}.$$

The walk’s starting triangle is the triangle that contains ${\left(x_{t-1},y_{t-1},z_{t-1}\right)}^{T}$. The *z*-component is hereafter ignored, as it is defined by the mesh’s triangles, which denote the walkable floor. Hereafter, (4) is used to determine the walk’s destination in *x* and *y* only. Whether the newly obtained destination ${\left(x_{t},y_{t}\right)}^{T}$ is actually reachable from the start ${\left(x_{t-1},y_{t-1}\right)}^{T}$ can be determined by checking if there is a way from the starting triangle towards some other, nearby triangle that contains these coordinates. If so, the discarded *z*-component $z_{t}$ is determined using the barycentric coordinates of ${\left(x_{t},y_{t}\right)}^{T}$ within a 2D projection of the triangle which the position belongs to, and applying them to the original 3D triangle. This can be though of walking along a 2D floor, and determining the floor’s altitude for the 2D destination. If this destination is unreachable, e.g., due to walls or other obstacles, different handling strategies are required. Simply trying again might be a viable solution, as uncertainty induced by T and S will yield a slightly different destination that might be reachable. Increasing $\sigma_{\mathrm{step}}$ and $\sigma_{\mathrm{turn}}$ for those cases might also be a viable choice. Likewise, just using some random position, omitting heading/steps might be viable as well.

The detected steps $n_{\mathrm{steps}}$ and the heading change Δθ used within above transition are obtained by the smartphone’s IMU. For the change in heading, we first need to rotate the gyroscope’s readings onto the east-north-up frame using a suitable rotation matrix, to determined, what the readings would look like, if the smartphone was placed parallel to the ground. The matrix is thus used to undo the rotation introduced by the pedestrian holding the phone. This rotation matrix is given by the matrix that rotates the current gravity readings from the accelerometer to the ${\left(0,0,9.81\;m\;s^{-2}\right)}^{T}$ vector. After applying the matrix to the gyroscope’s readings, the pedestrian’s change in heading (yaw) is given by integrating over the gyroscope’s *z*-axis [2]. It should be noted, that especially for cheap IMUs, as they can be found in most smartphones, the matrix has to be updated at very short intervals of one or two seconds to preserve good results [48].

To receive the number of steps, we use a very simple step detection based on the accelerometer’s magnitude. For this, we calculated the difference between the average magnitude over the last 200 ms and the gravity vector. If this difference is above a certain threshold (>$0.32\;m\;s^{-2}$), a step is detected. To prevent multiple detections within an unrealistic short interval, we block the complete process for 250 ms [49]. Of course, there are much more advanced methods as surveyed in [48], however this simple method has served us very well in the past.

## 5. Evaluation

The probability density of the state evaluation in (1) is given by

(5) $$p\left(o_{t}|q_{t}\right)=p{\left(o_{t}|q_{t}\right)}_{\mathrm{wifi}}\;p{\left(o_{t}|q_{t}\right)}_{\mathrm{act}},$$

where every component refers to a probabilistic sensor model which are statistical independent. The barometer and accelerometer readings are used to determine the current activity Ω, which is then evaluated using $p{\left(o_{t}|q_{t}\right)}_{\mathrm{act}}$. Absolute positioning information is given by $p{\left(o_{t}|q_{t}\right)}_{\mathrm{wifi}}$ for Wi-Fi.

### 5.1. Wi-Fi

As stated in Section 2, we use the smartphone’s Wi-Fi component to provide an absolute location estimation based on a comparison between recent RSSI measurements of nearby AP’s and signal strength predictions. The probability given those measurements $s_{\mathrm{wifi}}$ and a prediction, corresponding to a well-known location $\rho ={\left(x,y,z\right)}^{T}$ provided by $q_{t}$, can thus be written as

(6) $$p{\left(o_{t}|q_{t}\right)}_{\mathrm{wifi}}=p\left(s_{\mathrm{wifi}}|\rho \right)=\prod_{s_{i}\in s_{\mathrm{wifi}}}p\left(s_{i}|\rho \right),\;\rho \in R^{3}.$$

We assume a statistical independence between the respective AP’s. The comparison between a single RSSI measurement $s_{i}$ and the reference is given by

(7) $$p\left(s_{i}|\rho \right)=N\left(s_{i}|\mu_{i,\rho },\sigma_{\mathrm{wifi}}^{2}\right)\;,$$

where $\mu_{i,\rho }$ denotes the (predicted) signal strength for the AP identified by *i*, regarding the location ρ. A certain noise is allowed by the corresponding standard deviation $\sigma_{\mathrm{wifi}}$. Within this work $\mu_{i,\rho }$ is calculated by a compromise between the log-distance model and the wall-attenuation factor model [27], as presented in [6].

In contrary to its name the model only considers floors/ceilings, as including walls demands for costly intersection tests to determine all walls along the signal’s line-of-sight. While including walls within the model would increase the accuracy of the model’s prediction [27,28], for many use-cases it is sufficient to just consider floors/ceilings, to reduce the performance impact when being used on smartphones.

Therefore, the prediction depends on the 3D distance *d* between the AP in question and the location ρ as well as the number of floors Δf between them:

(8) $$\mu_{\rho }=P_{0}-10\gamma \log_{10}\frac{d}{d_{0}}+\Delta f\beta$$

Here, $P_{0}$ is the AP’s signal strength measurable at a known distance $d_{0}$ (usually 1 m) and γ denotes the signals depletion over distance, which depends on the AP’s surroundings like walls and other obstacles. The attenuation per floor is given by β. For example, a viable choice for steel enforced concrete floors is β≈−8.0 dB [2]. Of course, Equation (8) needs to be calculated separately for every *i* and thus available AP. It should be noted, that we omitted the index *i* in Equation (8) for the sake of clarity and consistency with other literature.

The environmental parameters $P_{0}$, γ and β need to be known beforehand and often vary greatly between single AP’s. Nevertheless, for simplicity’s sake it is common practice to use some fixed empirically chosen values, the same for every AP. This might already provide enough accuracy for some use-cases and buildings, but fails in complex scenarios, as discussed in Section 1. Therefore, instead of using a pure empiric model, we deploy an optimization scheme to find a well-suited set of parameters ($\hat{\varrho },P_{0},\gamma ,\beta$) per AP, where $\hat{\varrho }={\left(x,y,z\right)}^{T}$ denotes the AP’s estimated position. The optimization is based on a set of reference measurements $s_{\mathrm{opt}}$ throughout the building, e.g., every 3 to 7 m centred within a corridor and between 1 and 4 references per room, depending on the room’s size. Compared to classical fingerprinting, where reference measurements are recorded on small grids between 1 to 2 m, this highly reduces their required number and thus the overall setup-time. Of course, their are fast fingerprinting solutions like [31] (cf. Section 2), which are able to record the reference measurements while walking on predefined paths. Nevertheless, such an approach would also be compatible with the Wi-Fi model presented here and is thus a valid topic for future work.

The target function to optimize the 6 model parameters for one AP is given by

(9) $$\left(\hat{\varrho },P_{0},\gamma ,\beta \right)=\underset{\hat{\varrho },P_{0},\gamma ,\beta }{arg min}\sum_{s_{i}\in s_{\mathrm{mac}}}{\left(s_{i}-\mu_{\rho }\right)}^{2},\;\mu_{\rho }=P_{0}-10\gamma \log_{10}\frac{∥\rho -\hat{\varrho }∥}{d_{0}}+\Delta f\beta .$$

Here, one reduces the squared error between reference measurements $s_{i}\in s_{\mathrm{mac}}$ with well-known location ρ and corresponding model predictions $\mu_{\rho }$ (cf. Equation (8)). Whereas $s_{\mathrm{mac}}$ is the subset of $s_{\mathrm{opt}}$ for the AP in question, identified by its MAC-adress. The number of floors between ρ and $\hat{\varrho }$ is again given by Δf. As discussed by [6], optimizing all 6 parameters, especially the unknown AP position $\hat{\varrho }$, usually results in optimizing a non-convex, discontinuous function. A promising way to deal with non-convex functions is using a genetic algorithm, which is inspired by the process of natural selection [50].

The here deployed algorithm starts with a initial population, that is uniformly sampled within predefined limits of the to-be-optimized parameters. The AP’s location $\hat{\varrho }$ must be within the building and is therefore limited by its size. $P_{0}$, γ and β are set within a sane interval around empirically chosen values. During each iteration, the best 25% of the population are kept. The remaining entries are then re-created by modifying the best entries with uniform random values within ±10% of the known limits. Inspired by *cooling* known from simulated annealing [51], the result is stabilized by narrowing the allowed modification limits over time and thus decrease in the probability of accepting worse solutions.

To further improve the results, we optimize a model for each floor of the building instead of a single global one, using only the reference measurements that belong to the corresponding floor. The reason for this comes from the assumptions made in Equation (8). Here, no walls are considered and thus we expect erroneous RSSI measurements for regions that are heavily shrouded, e.g., by steel-reenforced concrete or metallized glass. During evaluation, the *z*-value from ρ in Equation (7) is used to select the correct model for this location, what then provides the signal strength prediction. For example, if a pedestrian walks on a staircase and thus is in-between multiple storeys, the average prediction of all corresponding models is calculated instead.

Basically, any kind of wireless network which allows to measure RSSI can be used for the above. However, most buildings do not provide a satisfying and well covered Wi-Fi infrastructure, e.g., staircases or hallways are often neglected for office spaces. This applies in particular to historical buildings, as discussed in Section 1. To improve Wi-Fi coverage we are able to distribute a small number of simple and cheap Wi-Fi beacons. As beacons we use a WEMOS D1 mini, which is based on the ESP-8266EX Wi-Fi chip [52]. The building considered in this work has no Wi-Fi infrastructure at all, not even a single AP. Nevertheless, our method allows to distribute beacons in the whole building by simply plugging them into available power outlets.

### 5.2. Activity Recognition

To enable continuous floor changes we use a simple activity recognition based on the smartphone’s barometer and accelerometer. The method distinguishes between the following: standing, walking, walking up or walking down. For each sensor we define two fixed-sized windows: $\omega_{s}$ (short) and $\omega_{l}$ (long). As their naming suggests, the windows differ in size and thus in the number of raw sensor measurements they hold. Both windows are implemented as real-time queues. Therefore, if a new sensor measurement is added, the oldest entry will be removed.

**Figure 2:**
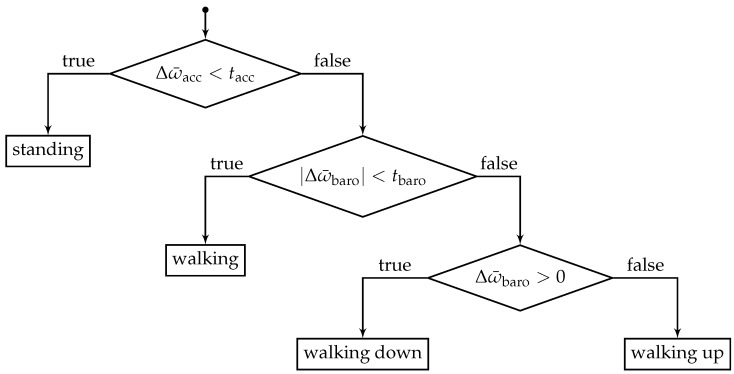
Decision tree describing the threshold-based activity recognition using the smartphone’s barometer and accelerometer measurements. The respective thresholds are given by $t_{\mathrm{acc}}$ and $t_{\mathrm{baro}}$. For each sensor the sigma of the arithmetic mean $\Delta \bar{\omega }={\bar{\omega }}_{l}-{\bar{\omega }}_{s}$ of two different fix-sized windows $\omega_{s}$ (short) and $\omega_{l}$ (long), holding a set of the most current sensor measurements, is calculated. The process updates with every incoming barometer reading.

To recognize the respective activities, we suggest a very simple threshold-based process. Defining a threshold $t_{\mathrm{acc}}$ for acceleration (m/s${}^{2}$) and $t_{\mathrm{baro}}$ for altitude (hPa). The corresponding activity is then detected as described by the decision tree given in Figure 2 for every incoming barometer measurement.

Here, $\Delta \bar{\omega }={\bar{\omega }}_{l}-{\bar{\omega }}_{s}$ and $\bar{\omega }$ provides the arithmetic mean of the respective windows and thus represents a moving average. We set $t_{\mathrm{acc}}=$ 0.015 m/$s^{2}$ and $t_{\mathrm{baro}}=$ 0.042 hPa. For both involved sensors we suggest to set the size of $\omega_{s}$ between 0.3 s and 0.6 s. Recognizing if the pedestrian is standing or walking requires less prior data, then climbing stairs. Therefore, $\omega_{l,\mathrm{acc}}$ is recommended between 1 s and 2 s, while $\omega_{l,\mathrm{baro}}$ between 3 s and 5 s. It should be noted, that the window size is a classic trade-off between flexibility and robustness. The larger the window, the slower changes become noticeable and vice versa. Of course, the above suggested values are dependent upon the particular requirements and used sensors. However, they should be valid for many modern commercially available smartphones.

The activity is now evaluated using $p{\left(o_{t}|q_{t}\right)}_{\mathrm{act}}$ by providing a probability based on whether the 3D location ρ of the state-in-question is on a staircase, in an elevator or on the floor. If the current activity Ω is recognized as “standing”, a ρ located on the floor results in a probability given by κ, otherwise 1−κ. The same applies to “walking up” and “walking down”, here a ρ located on one of the possible staircases or elevators provides κ and those who remain on the floor 1−κ. The likelihood for κ is chosen empirically. It is useful to find a reasonable value that is not too restrictive. In most cases, κ=0.75 provides good results by remaining enough room for erroneous classifications. A significant higher value like κ=0.99 could cause the system to be stuck on a staircase or to be unable to change floors.

## 6. Particle Filtering

As described earlier, we use a CONDENSATION particle filter to implement the recursive state estimator described in Section 3. A set of *N* particles is defined by ${\left\{X_{t}^{i},w_{t}^{i}\right\}}_{i=1}^{N}$, where $X_{t}^{i}$ is sampled based on the state transition $p(q_{t}|q_{t-1},o_{t-1})$. The weight $w_{t}^{i}$ is obtained by the probability density of the state evaluation $p(o_{t}|q_{t})$. A particle set approximates the posterior as follows:

(10) $$p\left(q_{t}|o_{1:t}\right)\approx \sum_{i=1}^{N}w_{t}^{i}\delta_{X_{t}^{i}}\left(q_{t}\right)\;,$$

where $\delta_{x_{0}}\left(x\right)$ denotes the Dirac delta mass located at $x_{0}$. As one can imagine, after a few iterations with continuously reweighting particles, the weight will concentrate on a few particles only. To handle this phenomenon of weight degeneracy, a resampling procedure is performed after every filter step [53].

### 6.1. State Estimation

Each particle is a realization of one possible system state, here the position of a pedestrian within a building. The set of all particles represents the posterior of the system. In other words, the particle filter naturally generates a sample based representation of the posterior. With this representation a point estimator can directly be applied to the sample data to derive a sample statistic serving as a “best guess”.

A popular point estimate, which can be directly obtained from the sample set, is the minimum mean squared error (MMSE) estimate. In the case of particle filters the MMSE estimate equals to the weighted-average over all samples, i.e., the sample mean

(11) $${\hat{q}}_{t}:=\frac{1}{W_{t}}\sum_{i=1}^{N}w_{t}^{i}X_{t}^{i},$$

where $W_{t}=\sum_{i=1}^{N}w_{t}^{i}$ is the sum of all weights. While producing an overall good result in many situations, it fails when the posterior is multimodal. In these situations the weighted-average estimate will find the estimate somewhere between the modes. It is expected that such a position between modes is extremely unlikely the position of the pedestrian. The real position is more likely to be found at the position of one of the modes, but virtually never somewhere between.

In the case of a multimodal posterior the system should estimate the position based on the highest mode. Therefore, the maximum a posteriori (MAP) estimate is a suitable choice for such a situation. A straightforward approach is to select the particle with the highest weight. However, this is in fact not necessarily a valid MAP estimate, because only the weight of the particle is taken into account. In order to compute the true MAP estimate the local density of the particles needs to be considered as well [54].

A computation of the probability density function of the posterior could solve the above, but finding such an analytical solution is an intractable problem, which is the reason for applying a sample representation in the first place. A feasible alternative is to estimate the parameters of a specific parametric model based on the sample set, assuming that the unknown distribution is approximately a parametric distribution or a mixture of parametric distributions, e.g., Gaussian mixture distributions. Given the estimated parameters the most probable state can be obtained from the parameterised density function. However, parametric models fail when the assumption does not fit the underlying model. For our application assuming a parametric distribution is too limiting as the posterior is changing in a non-predictable way over time.

On the other side a non-parametric approach directly obtains an estimate of the entire density function driven by the structure of the data. A classic non-parametric method is the kernel density estimator (KDE), where a kernel function with given bandwidth is placed at each particle to approximate the posterior. While the kernel estimate inherits all the properties of the kernel, usually it is not of crucial matter if a non-optimal kernel was chosen. As a matter of fact, the quality of the kernel estimate is primarily determined by the bandwidth. For our system we choose the Gaussian kernel in favour of computational efficiency.

The great flexibility of the KDE comes at the cost of a high computational time, which renders it unpractical for real time scenarios. The complexity of a naive implementation of the KDE is $O\left(MN\right)$, given by *M* evaluations and *N* particles as input size. A fast approximation of the KDE can be applied if the data is stored in equidistant bins as suggested by [55]. Computation of the KDE with a Gaussian kernel on the binned data becomes analogous to applying a Gaussian filter, which can be approximated by iterated box filter in $O\left(N\right)$ [5]. Our approximation scheme of the KDE is fast enough to estimate the density of the posterior in each time step. This allows us to recover the most prober state from occurring multimodal posterior.

### 6.2. Sample Impoverishment

**Figure 3:**
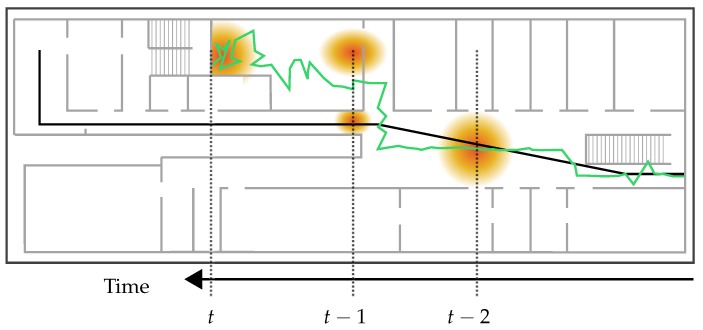
An example of the occurrence of sample impoverishment enhanced by a restrictive transition model that prevents sampling through walls. At time t−1 the approximated position (green line) drifts apart from the ground truth (black line) due to uncertain measurements. The posterior distribution is then captured within the room and not able to recover by itself [3].

As we have extensively discussed in [3], besides sample degeneracy, particle filters (and nearly all of its modifications) continue to suffer from another notorious problem: sample impoverishment. It refers to a situation, in which the filter is unable to sample enough particles into proper regions of the building, caused by a high concentration of misplaced particles. As described in Section 2, sample impoverishment is often a problem of environmental restrictions and system dynamics. An example using the so far presented approach can be seen in Figure 3. Due to uncertain measurements, the posterior distribution of the particle filter is captured within a room. Between time t−1 and *t*, the resampling step abandons all particles on the corridor and drawing new particles outside the room is not possible due to the restricted transition. At this point, standard filtering methods are not able to recover.

The simplest solution to handle sample impoverishment is by drawing a handful new particles randomly in the building. For this, we add a slight chance of 0.01% to the resampling step, so that every particle can be chosen for repositioning instead of the standard procedure. A new position for those particles is then drawn uniformely from the underlying mesh. It is obvious that this leads to a higher uncertainty and possibly a multimodal posterior distribution. Additionally, very uncertain absolute measurements, like attenuated Wi-Fi signals, can cause unpredictable jumps to such a newly drawn position, which would otherwise not be possible. Especially, methods using relative measurements like pedestrian dead reckoning are losing their importance. Nevertheless, this method is very easy to implement and we expect that the system should be able to recover from nearly every situation regardless of the cause.

A second method we suggest within this paper is a simplified version of our approach presented in [3]. Here, we used an additional, very simple particle filter to monitor if our primary (localization) filter suffers from sample impoverishment. If that is true, both filters are combined by exchanging particles among each other. This allows the primary filter to recover, while retaining prior knowledge. However, we believe that such a combination of two independent filters is not necessary for most scenarios and thus the resulting overhead can be avoided.

For the simplified version we draw a number of $N_{Q}$ locations $\rho_{Q}={\left(x,y,z\right)}^{T}$ uniformly from the underlying navigation mesh. Based on this locations we then approximate $p{\left(o_{t}|q_{t}\right)}_{\mathrm{wifi}}$ as described in Section 5.1. This results in a set of probabilities associated with $\rho_{Q}$, we call probability grid $Q_{t,\mathrm{wifi}}$. It is important to notice, that $Q_{t,\mathrm{wifi}}$ is newly created in every filter update, independently of the filter’s current particle set. Based on the grid, we are able to identify the areas where the (standalone) Wi-Fi model assumes the pedestrian to be most likely located. This often results in a multimodal representation of the probability density and thus multiple possible whereabouts. However, compared to the used particle filter, this representation enables us to monitor the complete building without any environmental restrictions and can thus be deployed as an indicator to detect sample impoverishment. If $Q_{t,\mathrm{wifi}}$ and the current posterior $p(q_{t}|o_{1:t})$ show a significant difference, we can assume that either the posterior got stuck and suffers from impoverishment or the Wi-Fi quality is low due to factors like attenuation or bad coverage. A good measure of how one probability distribution differs from a second is the well-established Kullback-Leibler divergence $D_{\mathrm{KL}}$ [3]. To calculate $D_{\mathrm{KL}}$, we need to sample densities from both probability density functions likewise. For the posterior we use the results provided by our kernel density estimation performed in the state estimation procedure, while $Q_{t,\mathrm{wifi}}$ is already in the desired form. The number of $N_{Q}$ is chosen empirically, depending on the size of the building and the detail of approximation required. Based on our experience $N_{Q}$ = 10,000 is a reasonable amount and keeps the workload stable for real time system.

Using $D_{\mathrm{KL}}$, we are now able to take countermeasures against sample impoverishment, depending on its size. However, those countermeasures will only work reliable if the Wi-Fi measurement noise is within reasonable limits. Attenuated or bad Wi-Fi readings are leading $D_{\mathrm{KL}}$ to grow, even if the posterior provides good results. For this, we introduce a Wi-Fi quality factor, enabling us to identify such situations. The quality factor is defined by

(12) $$q\left(o_{t}^{s_{\mathrm{wifi}}}\right)=\max\left(0,\min\left(\frac{{\bar{s}}_{\mathrm{wifi}}-l_{\min}}{l_{\max}-l_{\min}},1\right)\right)$$

where ${\bar{s}}_{\mathrm{wifi}}$ is the average of all signal strength measurements received from the observation $o_{t}^{s_{\mathrm{wifi}}}$. An upper and lower bound is given by $l_{\max}$ and $l_{\min}$. The quality factor is extensively discussed within [3,6].

Finally, we have all necessary tools to implement the second method to prevent impoverishment into the particle filter. For this, the state transition model is extended. Compared to the resampling step, as used by the first method, the transition $p(q_{t}|q_{t-1},o_{t-1})$ enables us to use prior measurements, which is obviously necessary for all Wi-Fi related calculations. As described in Section 4, our transition method only allows to sample particles at positions, that are actual feasible for a humans within a building e.g., no walking trough walls. If a particle targets a position which is not walk-able e.g., behind a wall, we deploy a strategy how to handle this. For example, drawing a new position within a very small, but reachable area around the particle’s current position. To prevent sample impoverishment we extend this transition strategy by making the reachable area depended upon $D_{\mathrm{KL}}$ and the Wi-Fi quality factor. Particles are thus drawn uniformly on a sub-region of the mesh, given by a radius $r_{\mathrm{sub}}=D_{\mathrm{KL}}\;\cdot \;q\left(o_{t}^{s_{\mathrm{wifi}}}\right)$. The sub-region consists of all walk-able and connected triangles within $r_{\mathrm{sub}}$, including stairs and elevators. This allows to increase the diversity of particles by the means of Wi-Fi, allowing to ignore any restrictions made by the system, as long as the difference between $Q_{t,\mathrm{wifi}}$ and the posterior is high. The subsequent evaluation step of the particle filter then reweights the particles, so that only those in proper regions will survive the resampling.

To further improve the method we give particles a chance of 0.01% to walk trough a nearby wall, if the destination is not outside. This enables to handle sample impoverishment more quickly in situations caused by environmental restrictions, even when the Wi-Fi quality is low. Especially in areas full of nooks an crannies, the vulnerability to errors should be decreased.

## 7. Experiments

As explained at the very beginning of this work, we wanted to explore the limits of the here presented localization system. By utilizing the proposed technology in a 13th century historic building, we created a challenging scenario not only because of the various architectural factors, but also because of its function as a museum. During all experiments, the museum was open to the public and had a varying number of 10 to 50 visitors while recording.

**Figure 4:**
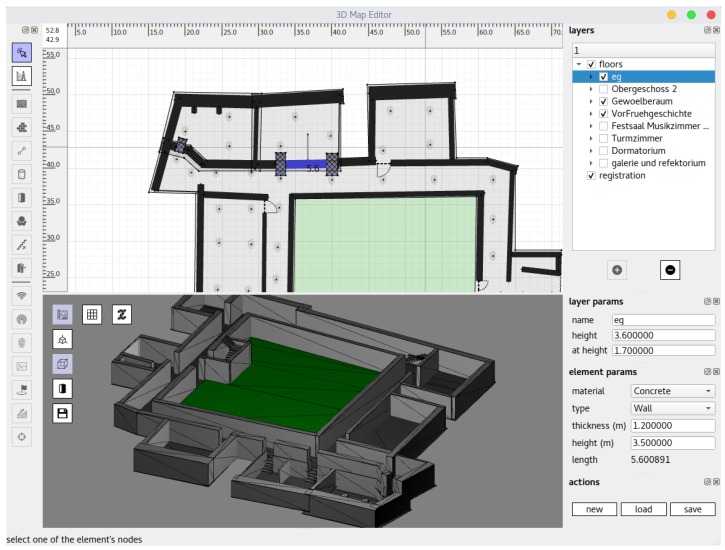
The 3D map editor we developed to create the floor plans. This screenshot shows the ground level of the building. The window is split into toolbar (left), layers (upper right), parameters of current selection (lower right), drawing mode (upper center) and 3D view (lower center).

The building consists of 6 different levels, which are grouped into 3 floors. Thus, the ceiling height is not constant over one floor and varies between 2.6 m to 3.6 m. While most of the exterior and ground level walls are made of massive stones, the floors above are half-timbered constructions. In the middle of the building is an outdoor area, which is only accessible from one side. The total walkable indoor area for a visitor is 2500 m^2^ in size. Due to objects like exhibits, cabinets or signs not all positions within the building were freely accessible. For the sake of simplicity we did not incorporate such knowledge into the floor plan. Thus, the floor plan consists only of walls, ceilings, doors, windows and stairs. It was created using our 3D map editor software (see Figure 4) based on architectural drawings from the 1980s. Our map editor is also used to automatically create the navigation mesh, which only takes a few seconds to compute.

As described in Section 5.1 we used 42 WEMOS D1 mini to provide a Wi-Fi signal coverage throughout the building. The distribution of the beacons on ground floor can be seen in Figure 7 (black dots) as well as the references (fingerprints) for optimization. The position of the beacons were chosen depending on available power sources. Care was taken to have at least two beacons in each room and a third beacon visible in an approximate radius of 10 m. Due to the difficult architecture and the extremely thick walls of the museum, we decided on this procedure, which explains the rather large number of 42 transmitters compared to modern buildings. Another reason for the high number of beacons is that we did not want to analyze the quality of the Wi-Fi signal coverage for further improvements, as this can be a very time-consuming task. In many areas of the building an improvement would not even be possible due to the lack of power sockets. The power sockets are located at different heights ranging from 0.2 m to 2.5 m. Consequently, there were no prior requirements on how a single beacon should be placed exactly and its position is dictated by the socket’s position. Considering all the above, the beacons were placed more or less freely and to the best of our knowledge.

A similar approach was chosen for placing the fingerprints. The positions of the fingerprints are set within our 3D map editor (see Figure 4) software by dragging the fingerprinting icon on the desired position or by entering the position manually. The reference points were placed every 3 m to 7 m from each other, however as can be seen in Figure 7 not necessarily accurate. As the optimization scheme does not require equally spaced reference points, doing so would result in superfluous effort. Furthermore, it is not easy to adopt the exact position to take the reference measurements in the building later on. Of course, this could be achieved with appropriate hardware (e.g., laser-scanner), but again, this requires more time and care, which in our opinion does not justify a presumably increased accuracy of some decimeters. Therefore, we accept the resulting inaccuracy between the (reference) position stored on the map and the actual position where the measurement took place, due to the enormous time saving.

Summing up the above, the following initial steps are required to utilize our localization system in a building:

1. Acquiring a blueprint or architectural drawing of the building including at minimum the walls and stairs of the respective floors.
2. Based on this 2D drawing, the floor plan is created manually using our 3D map editor (cf. Figure 4), comparable to software like Inkscape or FreeCAD.
3. If necessary, create or improve the Wi-Fi infrastructure by plugging in beacons to available power sockets and compose a whitelist of MAC-addresses of the involved access points or beacons.
4. Record the reference measurements based on the reference positions given in the floor plan.
5. The Wi-Fi model is optimized using the previously obtained reference measurements.
6. The navigation mesh is created automatically based on the before created floor plan as can be seen in Figure 1c.

**Figure 5:**
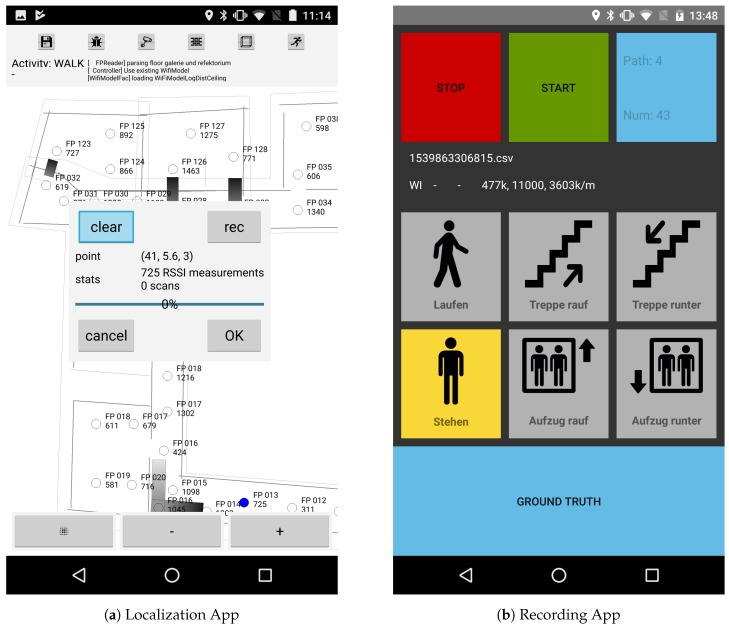
The two mobile applications developed for Android. The localization app in (**a**) is used to record the Wi-Fi reference measurements based on the positions provided by the floor plan. In this screenshot the dialog for recording them is visible. The app also implements the here presented approach and can thus be used for localization. However, for the utilized experiments we used a simpler client (**b**) allowing for user input like a ground truth or activity button.

For the building considered within this work, we were able to perform this steps in less then 160 min by a person, which is familiar with the system, and the janitor of the museum. Step 1 and 2 were conducted off-site. The blueprint was initially provided by the director of the museum as digital photography. Creating the floor plan including walls and stairs took us approximately 40 min and is then stored onto the smartphone after creation. Adding knowledge like semantic information such as room numbers would of course take additional time. All remaining steps were performed on-site using our smartphone app for localization, which can be seen in Figure 5a. As the museum did not provide any Wi-Fi infrastructure, we installed 42 beacons as explained above. With the help of the museum’s janitor, this step took only 30 min, as he was well aware of all available power outlets and also helped plugging them in. After that, 30 Wi-Fi scans were conducted and recorded for each of the 133 reference points using a Motorola Nexus 6 at the 2.4 GHz Wi-Fi band. This took approximately 25 s per point, as the Android OS restricts the scan rate. In total, this took 85 min, as all measurements were conducted using the same smartphone. The optimized Wi-Fi model and the mesh can be created automatically within a negligible amount of time directly on the smartphone, which then enables the pedestrian to start the localization. Of course, for the experiments conducted below several additional knowledge was obtained to evaluate the quality of the proposed methods and the overall localization error. Thus the above provided times were measured for a pure localization installation, as for example a customer would order, while the experiments were performed in a 2-day period. Nevertheless, we believe that an on-site setup-time of less than 120 min improves the practicability of the localization system, especially in commercial scenarios. In addition, the above steps do not require a high level of thoroughness in their execution or special knowledge about the details of the system, which should also allow unbiased persons to set up the system.

As mentioned, the here presented localization system was implemented as an Android App. It was written in high performant C++ code, enabling to run completely on the smartphone and thus not requiring any connection to a server. However, since the experiments required additional information to evaluate the methods, a second, simple application was developed to aid the recording of them (see Figure 5b). It implements the standard Android sensor functionalities and provides a minimalistic user interface so that even non-technical users can use it. As smartphones we used either a Samsung Note 2, Google Pixel One or Motorola Nexus 6. The computation of the state estimation as well as the Wi-Fi optimization are done offline using an Intel Core i7-4702HQ CPU with a frequency of 2.2 GHz running 8 threads on 4 cores and 16 GB main memory. An offline computation has practical advantages, such as easier evaluation of the results or shorter waiting times due to higher computing power. Nevertheless, Android app and offline application are both use the same C++ backend for localization.

The experiments are separated into five sections: At first, we discuss the performance of the novel transition model and compare it to our previous approach using a gridded graph structure. In Section 7.2 we have a look at Wi-Fi optimization and how the real AP positions differ from it. Following, we conducted several test walks throughout the building to examine the estimation accuracy (in meter) of the localization system and discuss the here presented solutions for sample impoverishment. In Section 7.4 the threshold-based activity recognition is evaluated, providing a detection rate for the test walks utilized before. Finally, the respective estimation methods are discussed in Section 7.5.

### 7.1. Transition

To compare our old graph-based model with our novel transition model presented within Section 4, we chose a simple scenario, in which a tester walks up and down a staircase several times. We used 5000 particles and did not perform an evaluation and resampling step to maintain the pure performance of the transition (step and heading). The number of particles was heuristically chosen and is based on our previous experience from other scenarios and competitions. In addition, it sill allows a stable performance of our Android app for localization. The filter starts at a fixed position and is updated after every newly recognized step. We set $\sigma_{\mathrm{step}}=0.1$ and $\sigma_{\mathrm{turn}}=0.1$ likewise. The cells of the gridded graph were 20 × 20 cm in size and the transition implemented as described in [8]. As discussed in Section 4, the mesh demands for a strategy, how to handle unreachable destinations. We chose a simple, yet effective strategy: whenever a destination is unreachable to a particle, it is removed and the last correct transitioning particle is duplicated. Of course, the graph does not require for such a rule, since particles are only allowed to move on nodes and search for neighbours.

**Figure 6:**
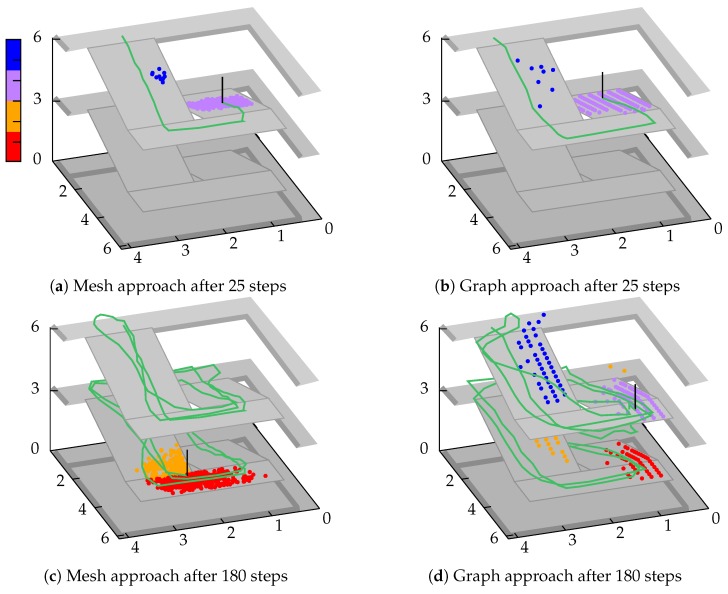
Simple staircase scenario to compare the old graph-based model with the new navigation mesh. All units are given in meter. The black line indicates the current position and the green line gives the estimated path until 25 or 180 steps, both using weighted average. The particles are colored according to their *z*-coordinate. A pedestrian walks up and down the stairs several times in a row. After 25 steps, both methods produce good results, although there are already some outliers (blue particles). After 180 steps, the outliers using the graph have multiplied, leading to a multimodal situation. In contrast, the mesh offers the possibility to remove particles that hit a wall and can thus prevent such a situation.

Figure 6a,b illustrate the results after 25 steps for each method. The particles are colored according to their z-coordinate and the walking path (green line) is estimated using weighted-average. It can be seen that both methods provide similar results. Due to the discrete grid structure, the purple particles on the graph scatter more strongly, while the mesh provides a truly continuous structure and thus a more compact representation. It is important to note that outliers have already appeared in both scenarios (blue particles). Due to the included sensor noise, they covered a too short distance for several times and thus the upcoming left turn leads upwards instead of downwards. Going straightforward to 180 steps, this phenomenon has multiplied for the graph (cf. Figure 6d), but not for the mesh (cf. Figure 6c). This is due to the above-mentioned strategy for the mesh. Compared to this approach, the graph is not able to remove any particles and thus they walk according to the recognized steps and heading changes, even if they theoretically hit a wall several times. After walking up and down twice, several particle groups have formed, which no longer allows an accurate position estimation.

Of course, a similar strategy could be developed for a graph. We have already shown how to identify the nodes nearest to walls in one of our previous works [8]. However, the limitation to walk in 45° angles as well as the discrete cell sizes lead to restrictions for small rooms, narrow hallways or bigger cells. For example walking through a door, would result in a strong reduction of differing particles. If the state evaluation is then used to assign weights to particles, the crucial problem of sample degeneracy often occurs. With a mesh, on the other hand, walkable destinations can also be located in a room behind a wall. In combination with the continues movement, this allows for a high versatility of particles even in such situations. Another method to fix the problems shown in Figure 6d, is by adding an activity recognition (walking up, down, straight) or to incorporate a barometer. Nevertheless, in most cases it is an advantage, if a sensor model delivers good results on its own, without further dependencies. For example, if a sensor is currently unavailable or damaged, the system is still able to provide proper results.

Besides the advantages the mesh offers, it also has a few disadvantages compared to the graph. The computation time has increased due to the calculation of reachable destinations. With the graph, only the direct neighbours are of interest, which can be implemented very efficiently using a tree structure. Further, the graph allows the easily store meta information on its nodes, for example Wi-Fi fingerprints or precalculations for shortest-path methods. This is more difficult using the mesh and requires the handling of baricentric coordinates.

### 7.2. Wi-Fi Optimization

**Figure 7:**
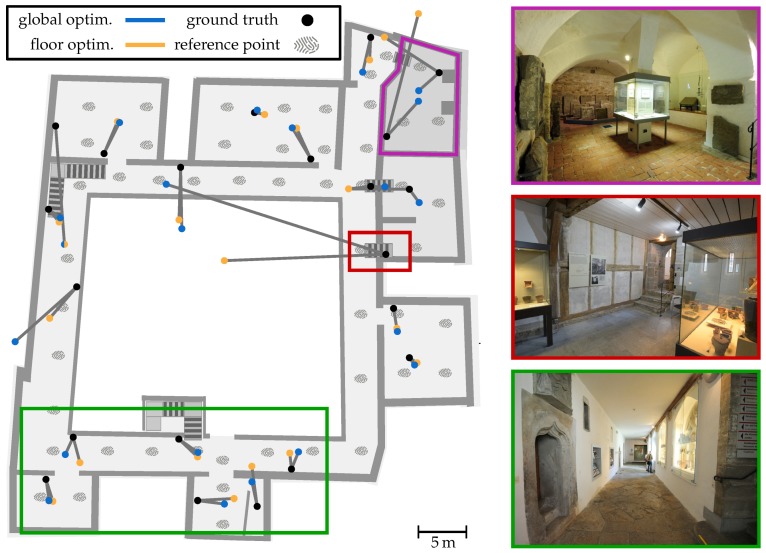
Ground level of the building in the xy-plane from above. Includes the locations of the reference points, the ground truth and the optimized APs. The grey line connects an AP with the corresponding optimization. The colored borders are areas of special interest and are discussed within the text. The corresponding pictures on the right side show the museum in these places.

Within all Wi-Fi observations, we only consider the beacons, which are identified by their well-known MAC address. Other transmitters like smart TVs or smartphone hotspots are ignored as they might cause estimation errors. The references (fingerprints) we used to optimize the Wi-Fi model as well as the real position of the APs (black dot) can be seen in Figure 7 for ground level. For the complete building we defined 133 reference points for recording Wi-Fi scans. An evaluation of how the number of references affects the optimization can be found in [6]. Increasing their number improves the result only up to a certain factor, which is why we have made a reasonable compromise between recording time and accuracy by distributing them every 3 m to 7 m from each other. Each reference location was then scanned 30 times (≈25 s scan time) using a Motorola Nexus 6 at 2.4 GHz band only. The resulting measurements were grouped per physical transmitter and aggregated to form the average signal strength per transmitter. The real position of every installed beacon was measured using a laser scanner. This allows a comparison with the optimized AP positions, what can also be seen in Figure 7.

It illustrates the results of the global (blue) and the per-floor (orange) method for all AP’s installed to ground level. The respective optimized positions $\hat{\varrho }$ are connected by a grey line with the corresponding ground truth, providing the position error on the xy-plane. The average distance error (3D) between the AP’s real position and the optimized ones is 5.4 m (μ= 5.1) for the per-floor and 4.8 m (μ= 5.6) for global strategy. However, it is easy to see that the results are better in some areas (green) than in others (red and purple). While the green rectangle encloses an area that has a high number of APs with line-of-sight conditions, the APs in red and purple are shielded by very thick stone walls and have a lower number of reference points with direct visual contact (cf. Figure 7). The maximum position error for the global scheme is 25.3 m and 18.4 m for the per-floor one. Both are related to the AP in the red rectangle.

Of course, the position alone does not provide sufficient information of the overall performance, however it provides a good visual impression of how the optimization behaves. The overall optimization error is measured using the difference between model predictions and real-world RSSI values for each reference measurement. These differences can be positive or negative, which is why we indicate an absolute or signed error. The (absolute) optimization error of the respective strategies is 4.7 dB (μ= 3.8) for global and 2.6 dB (μ= 2.7) for per-floor in average. Again, the highest errors occur from APs within the red and purple area, whereby the local maxima for the signed difference are −31.4 dB and 17.5 dB for global and −12.7 dB and 13.4 dB for local. Thus, the per-floor optimization scheme provides a smaller overall error, whereby the positioning error is higher compared to the global one. The reason for the latter can be found within the purple area. It marks a vaulted cellar, that is 1.7 m deeper than ground level and connect by a narrow staircase. Here, RSSI measurements received from AP’s residing on the ground level are strongly attenuated due to the massive walls of the cellar. In contrast, measurements coming from the floor above are much less attenuated thanks to a much thinner ceiling. Since the per-floor scheme uses only references from the current floor in question, while the global scheme uses all available references and thus more meaningful information in this area. However, as the overall error suggests, this is not always an advantage, which we will see later on in the localization experiments. As mentioned above, some areas are heavily attenuated by big walls, what simply does not fit the used signal strength prediction model. As discussed in Section 2 and Section 5.1, we only consider ceilings within the model to avoid computational expensive wall intersection-tests. A far higher number of reference measurements in bad areas can therefore only increase the accuracy to a limited extent, whereas increasing the number of reference points could compensate for this, however requires additional setup time, what is then contrary to a fast deploy time. Nevertheless, by optimizing all parameters ($\hat{\varrho }$, $P_{0}$, γ and β) the system provides far better localization results compared to using the AP’s real positions with empirical values or even optimized values only for $P_{0}$, γ and β. The reason for this is obvious. The optimized parameters fit the (unrealistic) signal strength prediction model much better than the real ones and thus provide for a smaller error between measured RSSI and predicted RSSI. Since walls are ignored by the model, optimizing the position of the access points can compensate for the resulting effects. This is also the reason why the optimized positions of AP’s attached to walls always have a certain distance to them, as can be seen in Figure 7. A more realistic model would not only mean an overall improvement of the results, but also a further approximation to the real conditions in the building. It is to be expected that the estimated positions of the access points will then approach the ground truth. Further evaluations and discussions regarding the here used optimization can be found in [6].

### 7.3. Localization Error

**Figure 8:**
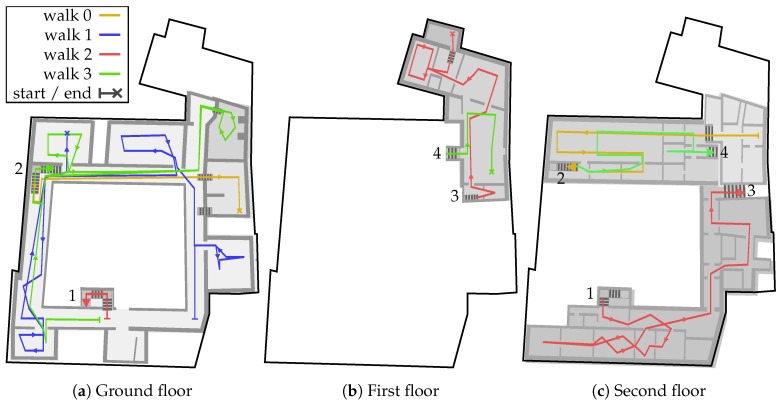
All conducted walks within the building. The arrows indicate the running direction and a cross marks the end. For a better overview we have divided the building into three floors, which are connected by four stairs (numbered 1–4). However, each floor consists of different high levels. They are separated from each other by different shades of grey, dark is lower than light.

The 4 chosen walking paths can be seen in Figure 8. Walk 0 is 152 m long and took about 2.30 min to walk. Walk 1 has a length of 223 m and Walk 2 a length of 231 m, both required about 6 min to walk. Finally, walk 3 is 310 m long and takes 10 min to walk. Each of the single walks was carried out by 4 different male testers using either a Samsung Note 2, Google Pixel One or Motorola Nexus 6 for recording the measurements. All in all, we recorded 28 distinct measurement series, 7 for each walk. The picked walks intentionally contain erroneous situations, in which many of the above treated problems occur. A walk is indicated by a set of numbered markers, fixed to the ground. Small icons on those markers give the direction of the next marker and in some cases provide instructions to pause walking for a certain time. The intervals for pausing vary between 10 s to 60 s. The ground truth is then measured by recording a timestamp while passing a marker. For this, the tester clicks a button on the smartphone application. Between two consecutive points, a constant movement speed is assumed. Thus, the ground truth might not be 100% accurate, but fair enough for error measurements. The approximation error is then calculated by comparing the interpolated ground truth position with the current estimation [4]. An estimation on the wrong floor has a great impact on the location awareness of an pedestrian, but only provides a relatively small error. Therefore, errors in *z*-direction are penalized by tripling the *z*-value.

For each walk we deployed 100 runs using 5000 particles and set $N_{\mathrm{eff}}=0.85$ for resampling. Instead of an initial position and heading, all walks start with a uniform distribution (random position and heading) as prior. The overall localization results can be see in Table 1. Here, we differ between the respective anti-impoverishment techniques presented in Section 6.2. The simple anti-impoverishment method is added to the resampling step and thus uses the transition method presented in Section 4. In contrast, the $D_{\mathrm{KL}}$-based method extends the transition and thus uses a standard cumulative resampling step. We set $l_{\max}=$ −75 dBm and $l_{\min}=$ −90 dBm. For a better overview, we only used the KDE-based estimation, as the errors compared to the weighted-average estimation differ by only a few centimeter.

The same applies for an accuracy comparison between the graph-based model and the navigation mesh as part of the overall system. Both provide very similar localization errors regarding the conducted walks. This is not a big surprise, as the accuracy of the pedestrian’s position based on the estimated state and thus the complete posterior density (weighted particle set). It is obvious, that choosing a graph with a grid-size of e.g., 2 × 2 m would worsen the results. This leads to the statement, that the approximation error of walking alongside the edges of a (reasonable sized) gridded graph is small enough that it has no significant influence on the overall localization accuracy compared to a true continuous motion. Nevertheless, as shown in Section 7.1, the navigation mesh offers several major benefits by highly reducing the memory footprint.

**Figure 9:**
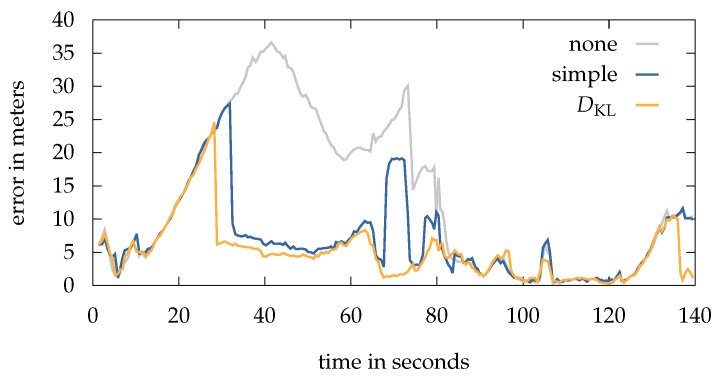
Error development over time of a single particle filter run of walk 0. Between 10 s and 24 s the Wi-Fi signal was highly attenuated, causing the system to get stuck and producing high errors. Both, the simple and the $D_{\mathrm{KL}}$ anti-impoverishment method are able to recover early. However, between 65 s and 74 s the simple method produces high errors due to the high random factor involved.

All walks, except for walk 1, suffer in some way from sample impoverishment. We discuss the single results of Table 1 starting with walk 0. Here, the pedestrians started at the top most level, walking down to the lowest point of the building. The first critical situation occurs immediately after the start. While walking down the small staircase, many particles are getting dragged into the room to the right due to erroneous Wi-Fi readings. At this point, the activity “walking down” is recognized, however only for a very short period. This is caused by the short length of the stairs. After this period, only a small number of particles changed the floor correctly, while a majority is stuck within the right-hand room. The activity based evaluation $p{\left(o_{t}|q_{t}\right)}_{\mathrm{act}}$ prevents particles from further walking down the stairs, while the resampling step mainly draws particles in already populated areas. In 10% of the runs using none of the anti-impoverishment methods, the system is unable to recover and thus unable to finish the walk somewhere near the correct position or even on the same floor. Yet, the other 90% of runs suffer from a very high error. Only by using one of the here presented methods to prevent impoverishment, the system is able to recover in 100% of cases. Figure 9 compares the error over time between the different methods for an exemplary run. The above described situation, causing the system to stuck after 10 s, is clearly visible. Both, the simple and the $D_{\mathrm{KL}}$ method are able to recover early and thus decrease the overall error dramatically. Between 65 s and 74 s the simple method produces high errors due to some uncertain Wi-Fi measurements coming from an access point below, causing those particles who are randomly drawn near this AP to be rewarded with a very high weight. This leads to newly sampled particles in this area and therefore a jump of the estimation. The situation is resolved after entering another room, which is now shielded by stone walls instead of wooden ones. Walking down the stairs at 80 s does also recover the localization system using none of the methods.

A similar behavior as the above can be seen in walk 3. Without a method to recover from impoverishment, the system lost track in 100% of the runs due to a not detected floor change in the last third of the walk. By using the simple method, the overall error can be reduced and the impoverishment resolved. Nevertheless, unpredictable jumps of the estimation are causing the system to be highly uncertain in some situations, even if those jumps do not last to long. Only the use of the $D_{\mathrm{KL}}$ method is able to produce reasonable results.

**Figure 10:**
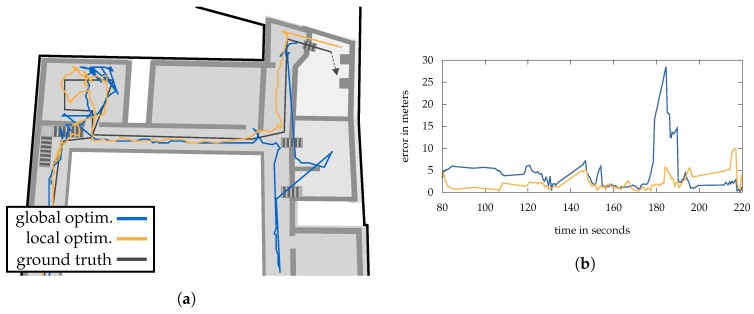
(**a**) A small section of walk 3. Optimizing the system with a global Wi-Fi optimization scheme (blue) causes a big jump and thus high errors. This happens due to highly attenuated Wi-Fi signals and inappropriate Wi-Fi parameters. We compare this to a system optimized for each floor individually (orange), resolving the situation a producing reasonable results; (**b**) Error development over time for this section. The high error can be seen at 190 s.

As described in Section 5.1, we use a Wi-Fi model optimized for each floor instead of a single global one. A good example why we do this, can be seen in Figure 10a, considering a small section of walk 3. Here, the system using the global Wi-Fi model makes a big jump into the right-hand corridor and requires 5 s to recover. This happens through a combination of environmental occurrences, like the many different materials and thus attenuation factors, as well as the limitation of the here used Wi-Fi model, only considering ceilings and ignoring walls. Following, AP’s on the same floor level, which are highly attenuated by 2 m thick stone walls, are neglected and AP’s from the floor above, which are only separated by a thin wooden ceiling, have a greater influence within the state evaluation process. Of course, we optimize the attenuation per floor, but at the end this is just an average value summing up the AP’s surrounding materials. Therefore, the calculated signal strength predictions do not fit the measurements received from the above in a optimal way. In contrast, the model optimized for each floor only considers the respective AP’s on that floor, allowing to calculate better fitting parameters. A major disadvantage of the method is the reduced number of visible AP’s and thus measurements within an area. This could lead to an underrepresentation of AP’s for triangulation. Such a scenario can be seen in Figure 10b between 200 s and 220 s, where the pedestrian enters an isolated room. Only two AP’s provide a solid signal within this area, leading to a higher error, while the global scheme still receives RSSI readings from above.

Looking at the results of Table 1 again, it can be seen that the $D_{\mathrm{KL}}$ method is able to improve the results in three of the four walks. Those walks have in common, that they suffer in some way from sample impoverishment or other problems causing the system to stuck. The only exception is walk 1. It was set up to provide a challenging scenario, leading to as many multimodalities as possible. We intentionally searched for situations in which there was a great chance that the particle set would separate, e.g., by providing multiple possible whereabouts through crossings or by blocking and thus separating a straight path with objects like movable walls. Similar to the other walks, we added different pausing intervals of 10 s to 60 s. This helps to analyse how the particles behave in such situations, especially in this multimodal setting.

**Figure 11:**
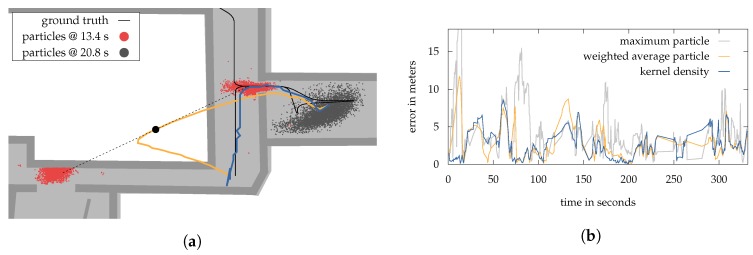
(**a**) Occurring bimodal distribution caused by uncertain measurements in the first 13.4 s of walk 1. After 20.8 s, the distribution gets unimodal. The weigted-average estimation (orange) provides a high error compared to the ground truth (solid black), while the KDE approach (blue) does not; (**b**) Error development over time for the complete walk. From 230 s to 290 s to pedestrian was not moving.

Besides uncertain measurements, one of the main sources for multimodalities are restrictive transition models, e.g., no walking through walls. As shown in Section 6.2, the $D_{\mathrm{KL}}$ method compares the current posterior $p(q_{t}|o_{1:t})$ with the probability grid $Q_{t,\mathrm{wifi}}$ using the Kullback-Leibler divergence and a Wi-Fi quality factor. Environmental restriction like walls are not considered while creating $Q_{t,\mathrm{wifi}}$, that is why the grid is not effected by a transition-based multimodal setting. Given accurate Wi-Fi measurements, it is therefore very likely that $Q_{t,\mathrm{wifi}}$ represents a unimodal distribution, even if the particles got separated by an obstacle or wall. This leads to a situation, in which posterior and grid differ. As a result, the radius $r_{\mathrm{sub}}$ increases and thus the diversity of particles. We are able to confirm the above by examining the different scenarios integrated into walk 1. For this, we compared the error development with the corresponding radius $r_{\mathrm{sub}}$ over time. In situations where the errors given by the $D_{\mathrm{KL}}$ method and the simple method differ the most, $r_{\mathrm{sub}}$ also increases the most. Here, the radius grows to a maximum of $r_{\mathrm{sub}}=$ 8.4 m, using the same measurement series as in Figure 11b. In contrast, a real sample impoverishment scenario, as seen in walk 0 (cf. Figure 9), has a maximum radius of 19.6 m. Nevertheless, such an slightly increased diversity of 8.4 m is enough to influence the estimation error of the $D_{\mathrm{KL}}$ in a negative way (cf. walk 1 in Table 1). Ironically, this is again some type of sample impoverishment, caused by the aforementioned environmental restrictions not allowing particles inside walls or other out of reach areas.

### 7.4. Activity Recognition

In order to evaluate the activity recognition, a test person had to press a button according to their current state of motion, namely standing, walking, walking up, walking down, elevator up and elevator down (cf. Figure 5b). As the building does not have an elevator, this state is ignored in the following. Whether a state needs to be changed was indicated by small symbols on the ground truth markers. This experiment is based on the same measurement series as Section 7.3. As the activity recognition uses moving averages, the detection suffers from a certain lag, determined by the window size. Thus, comparing each activity that is newly calculated with incoming barometer measurements to the ground truth at the current timestamp, would result in a rather low detection rate for the respective activities. In addition, only a fraction of a test path consists of the change of an activity, since the testers were walking most of the time. This would bias an overall detection rate.

In order to be able to make a statement about the quality, we first determined the average (time) lag of the conducted walks and then shifted the calculated data accordingly. This does not allow a perfect, but at least fair examination of the detection rate. The lag is given as the (absolute) difference between the timestamp, the activity swaps in ground truth (e.g., from standing to walking), and the first timestamp of an interval, given by the size of $\omega_{s}$, holding the same activity, given by our recognition method. This provides in an average lag of 2.96 s and a standard deviation of 1.09 s over all walks. The resulting detection rates can be seen in Table 2. They were calculated by dividing the number of correctly detected activities with the number of activities given by the ground truth. The first thing to notice is the bad recognition rate of standing, especially in comparison to the others. A major impact on this, is the fact, that we encourage the testers to behave as natural as possible, i.e., like a normal visitor of the museum. As a result, they often turned around or a took a few small steps within the standing sequences, to look at the exhibits.

This behavior is not mapped by the ground truth. In addition, using only acceleration for detection might be a bad choice in the first place, as moving the phone, e.g., by putting it in the trouser pocket, will exceed the threshold. At the end, this leads to the general question, on how to define standing. Is it a complete standstill or should it allow for a certain degree of freedom? The answer of this question often depends on the respective scenario. As for the museum, in which visitors often stand in front of exhibits or only move within a small area, the results for detecting the standing activity are not sufficient and a more advanced approach should be considered.

In contrast, the detection rates for walking up or down are clearly better. With only a single exception in walk 3 (cf. Section 7.3), the approach makes it possible to direct particles smoothly over stairs. Due to the lag, however, there is a noticeable delay of the estimation when entering the staircase. During this short period of time, the particles gather in front of the staircase, as they only receive a corresponding weighting when the activity changes. In buildings with many successive stairs, this could lead to further problems, as it affects the PDR-based movement of the particles. Nevertheless, in a scenario such as the present, the absolute positioning of Wi-Fi should compensate this. Finally, the detection rates did not allow to derive the concrete smartphone or the user, which indicates the generality of the approach.

### 7.5. Estimation

As mentioned before, the single estimation methods (cf. Section 6.1) only vary by a few centimetres in the overall localization error. That means, they differ mainly in the representation of the estimated locations. More easily spoken, in which way the estimated path is drawn and thus presented to the user. Regarding the underlying particle set, different shapes of probability distributions need to be considered, especially those with multimodalities.

The main advantage of a KDE-based estimation is that it provides the “correct” mode of a density, even under a multimodal setting (cf. Section 6.1). That is why we again have a look at walk 1. A situation in which the system highly benefits from this is illustrated in Figure 11a. Here, a set of particles splits apart, due to uncertain measurements and multiple possible walking directions. Indicated by the black dotted line, the resulting bimodal posterior reaches its maximum distance between the modes at 13.4 s. Thus, a weighted-average estimation (orange line) results in a position of the pedestrian somewhere outside the building (light green area). The ground truth is given by the black solid line. The KDE-based estimation (blue line) is able to provide reasonable results by choosing the “correct” mode of the density. After 20.8 s the setting returns to be unimodal again. Due to a right turn the lower red particles are walking against a wall and thus punished with a low weight.

Although, situations as displayed in Figure 11a frequently occur, the KDE-estimation is not able to improve the overall estimation results. This can be seen in the corresponding error development over time plot given by Figure 11b. Here, the KDE-estimation performs slightly better then the weighted-average, however after deploying 100 runs of the particle filter, the difference becomes insignificant. It is obvious, that the above mentioned “correct” mode, not always provides the lowest error. In some situations the weighted-average estimation is often closer to the ground truth. Within our experiments this happened especially when entering or leaving thick-walled rooms, causing slow and attenuated Wi-Fi signals. While the system’s dynamics are moving the particles outside, the faulty Wi-Fi readings are holding back a majority by assigning corresponding weights. Only with new measurements coming from the hallway or other parts of the building, the distribution and thus the KDE-estimation are able to recover.

**Figure 12:**
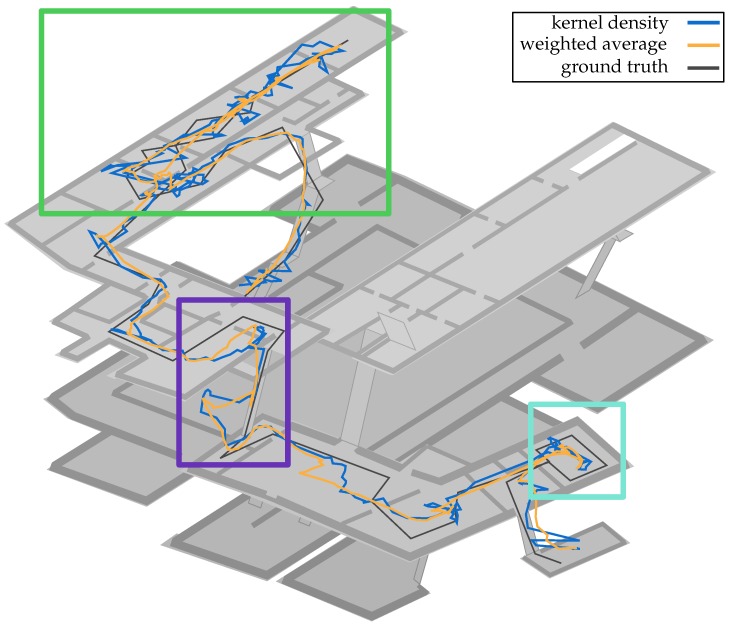
Estimation results of walk 2 using the KDE method (blue) and the weighted-average (orange). While the latter provides a more smooth representation of the estimated locations, the former provides a better idea of the quality of the underlying processes. In order to keep a better overview, the top level of the last floor was hidden. The colored rectangles mark interesting areas. Within the green rectangle, the above mentioned differences between the two methods are clearly visible. The purple rectangle displays a situation in which a sample impoverishment was successfully resolved. The teal rectangle marks an area were both methods do not provide sufficient results.

This leads to the conclusion, that a weighted-average approach provides a more smooth representation of the estimated locations and thus a higher robustness. A comparison between both methods is illustrated in Figure 12 using a measuring sequence of walk 2. We have highlighted some interesting areas with colored rectangles. The greatest difference between the respective estimation methods can be seen inside the green rectangle, the gallery wing of the museum. While the weighted-average (orange) produces a very straight estimated path, the KDE-based method (blue) is much more volatile. This can be explained by the many small rooms that pedestrians pass through. The doors act like bottlenecks, which is why many particles run against walls and thus are either drawn on a new position within a reachable area (cf. Section 6.1) or walk along the wall towards the door. This causes a higher uncertainty and diversity of the posterior, what is more likely to be reflected by the KDE method than by the weighted-average. Additionally, the pedestrian was forced seven times to look at paintings (stop walking) between 10 s and 20 s, just in this small area. Nevertheless, even if both estimated paths look very different, they produce similar errors.

The purple rectangle displays a situation in which a sample impoverishment was successfully resolved. Due to a poorly working AP, in the lower corner of the big room the pedestrians passes before walking down the stairs, the majority of particles is dragged into the upper right corner of that room and unable to walk down. By allowing some particles to walk through the wall and thus down the stairs, the impoverishment could be dissolved. The KDE-based estimation (blue line) illustrates this behavior very accurate. At first, the pedestrian’s position is estimated in the area around the corner of the room, after the impoverishment was recognized, the estimated path is then crossing the wall, enabling the floor change. However, as could be seen in Figure 7, before the here presented methods are able to resolve sample impoverishment, the error and thus the radius $r_{\mathrm{sub}}$ increase in time as the system got stuck. This can take up to a few seconds, in which the pedestrian has continued walking and is thus ahead of the current position estimation. As the first particles are newly drawn into more proper regions, the system starts to recover, still remaining in an uncertain state as the particle set is split apart. After a few filter updates, especially resampling steps, the system returns back to a more stable state. Both, the time in which the system was uncertain as well as the lag to the real position of the pedestrian lead to problems at the end of the staircase (left corner of the purple rectangle). While the pedestrian has already completely descended the stairs, the activity changes from walking down to walking. Since the majority of the particles are still on the stairs, they are thus considered to be less likely than particles on a floor. As there are only a few particles on the floors, some below and some above, the estimation is calculated somewhere in between, which finally explains the increased error in this area.

Another situation in which the estimated paths do not provide sufficient results can be seen inside the teal rectangle. The room is very isolated from the rest of the building, which is reflected by the fact that only 3 AP’s are detected. The pedestrians have been asked to cross the room at a quick pace, leading to a higher step rate and therefore update rate of the filter. The results within this area lead to the assumption, that even if Wi-Fi has a bad coverage, it influences the estimation results the most. The PDR based transition alone is able to walk alongside the ground truth in an accurate manner. However, this is of course only true if we consider this area individually, without the rest of the walk due to the accumulating bias of the relative sensors involved. We hope to further improve such situations in future work by enabling the transition step to provide a weight to particles that walk very likely, especially in situation where Wi-Fi provides bad readings.

To summarize, the KDE-based approach for estimation is able to resolve multimodalities. It does not provide a smooth estimated path, since it depends more on an accurate sensor model than a weighted-average approach, but is suitable as a good indicator about the real performance of a sensor fusion system. At the end, we only used the KDE approach to provide a global maxima, even though it opens a wide range of other possibilities for finding a best estimate. A detailed examination of the runtime performance of the used estimation methods in comparison to the state-of-the-art can be found in [5].

## 8. Conclusions

Within this work we provided an extensive overview of our smartphone-based indoor localization system, providing both, previous advances and novel contributions. The thorough evaluation demonstrated the good performance under multiple scenarios within a complex environment. The system is able to handle problems like sample impoverishment and multimodal densities, occurring through the use of a particle filtering scheme. Based on the promising results achieved in this challenging scenario, we believe that our solution can be adapted to various public buildings and environments, resulting in a generally usable solution for self-localization of pedestrians using smartphones. Previous versions of the system have already proven themselves in other, more modern buildings, which supports this claim to general use.

Thanks to the novel contributions presented, we have been able to further increase the robustness and accuracy of the system. To a large extent, this was achieved by using the navigation mesh. It allows to map continuous movements and enables to reduce the map sizes to only a few megabytes for a complete building. The problem of sample impoverishment can be addressed easily by incorporating the here presented method onto the state transition of the particle filter. In combination with the threshold-based activity recognition, both methods further enhance the robustness. Given the improvements above and those achieved in previous works, the main advantage of our approach is its suitability for practical use. Compared to other state-of-the-art solutions, the setup time is only a few hours and does not require any expert knowledge or hardware. The system should require as little manual effort as possible. This is mainly achieved by the optimization scheme, providing all necessary parameters for the Wi-Fi model. The localization runs solely an a commercial smartphone, thus no connection to a server or the Wi-Fi infrastructure is required.

Nevertheless, there is still room for further improvements and future work. Through the change from a graph to a mesh, we lost the ability to easily find the shortest path for navigation purposes as described in [8]. By means of barycentric coordinates, this should however be easily adaptable to the triangular structure. The threshold-based activity recognition is not able to distinguished between different types of elevation, namely elevator, escalator and stairs. Especially in buildings where elevators pass many floors, the transition fails to move particles in the according speed. Here, we need to incorporate special environmental knowledge about elevators and escalators or again integrate a probabilistic sensor model for the barometer as already done in previous works [2].

A crucial point to further increase the accuracy of the system is the choice of the signal strength prediction model. Currently we consider only the attenuation per floor, however by including information about walls and other obstacles, we should be able to decrease the error at the cost of additional computations. Instead of providing those additional environmental informations by manual measurements, the optimization scheme could be used to approximate the respective model and material parameters. Special data-structures for pre-computation combined with online interpolation might then be a viable choice for utmost accuracy that is still able to run on a commercial smartphone in real-time.

Finally, the approximation scheme for the KDE is capable of offering completely new possibilities when handling particle sets. Within this paper we used it to find the real global maxima for a state estimation and to accurately calculate the Kullback-Leibler divergence. However, many other estimation schemes are thinkable, for example a trajectory based one, with multiple path-hypotheses, each weighted based on a-priori knowledge. The KDE approach could also be used to develop better suited resampling techniques, by enabling to draw particles from the underlying density, instead of just reproducing known owns.

## Figures and Tables

**Table 1:** Overall localization results in meter using the different impoverishment methods. For estimation we used the KDE-based method, as the errors compared to the weighted-average differ by only a few centimeter. The results are presented given the average positioning error $\bar{x}$, the standard deviation $\bar{\sigma }$ and the 75%-quantil of positioning errors ${\tilde{x}}_{75}$.

	None				Simple				$D_{\mathrm{KL}}$		
	$\bar{x}$	$\bar{\sigma }$	${\tilde{x}}_{75}$		$\bar{x}$	$\bar{\sigma }$	${\tilde{x}}_{75}$		$\bar{x}$	$\bar{\sigma }$	${\tilde{x}}_{75}$
walk 0	13.4 m	11.2 m	22.6 m		7.1 m	6.6 m	9.4 m		5.8 m	4.9 m	7.3 m
walk 1	3.2 m	2.4 m	4.1 m		3.2 m	2.6 m	4.0 m		3.8 m	3.2 m	4.6 m
walk 2	8.3 m	4.1 m	10.9 m		3.6 m	2.3 m	4.9 m		3.6 m	2.3 m	4.8 m
walk 3	7.0 m	5.9 m	13.5 m		5.4 m	4.7 m	7.7 m		4.8 m	4.3 m	6.5 m

**Table 2:** The resulting detection rates provided by the activity recognition for all conducted walks. As the method suffers from a (time) lag, caused by the used moving average, we shifted the measured activity according to the average lag over all walks (2.96 s). Some cells of the table are empty, because the respective walk did not require this activity.

	Standing	Walking	Walking up	Walking down	Overall
walk 0	65.6%	80.9%	-	84.8%	78.4%
walk 1	49.9%	84.1%	-	-	67.5%
walk 2	57.4%	83.5%	83.5%	82.1%	71.7%
walk 3	45.7%	77.5%	85.1%	77.8%	61.3%
overall	51.4%	81.5%	84.3%	82.1%	67.9%
